# Synergistic Activation of Electric Furnace Ferronickel Slag by Mechanical Grinding and Chemical Activators to Prepare Cementitious Composites

**DOI:** 10.3390/ma17061247

**Published:** 2024-03-08

**Authors:** Yanjun Jiang, Xuqin Duan, Bohua Li, Shuaiyu Lu, Tong Liu, Yunyun Li

**Affiliations:** 1 School of Civil and Resource Engineering, University of Science & Technology Beijing, Beijing 100083, China; m202320093@xs.ustb.edu.cn (Y.J.); m202320108@xs.ustb.edu.cn (S.L.); m202220081@xs.ustb.edu.cn (T.L.); lyyustb@163.com (Y.L.); 2 State Key Laboratory for Efficient Mining of Metal Mines, University of Science & Technology Beijing, Beijing 100083, China

**Keywords:** electric furnace ferronickel slag, synergistic activation, cementitious composites, hydration mechanism

## Abstract

The use of electric furnace ferronickel slag (FNS) as a supplementary cementitious material is the current focus of research. This study investigates the effect of mechanical grinding and chemical additives on the activity excition of FNS, as well as the associated synergistic mechanisms. This study shows that the addition of triethanolamine (TEA) increases the fine-grained content in FNS powder, which facilitates the depolymerization of FNS and the early hydration of aluminum tricalcium. Furthermore, the addition of Ca(OH)_2_ raises the alkalinity of the cementitious system, which promotes the availability of Ca^2+^ ions and accelerates the hydration process, resulting in the generation of additional hydration products. The enhancement of late hydration of C_3_S by TEA and its combination with the secondary hydration of Ca^2+^ at high alkalinity are the pivotal factors to improve the strength of cementitious composite. A mixture of FNS and 0.03% TEA is subjected to grinding for 90 min, using the obtained micropowder which replaces 20% of the cement, and subsequently, after being excited with 3% Ca(OH)_2_, the FNS micropowder reaches the quality standards of S95 slag powder. It is worth remarking that the micropowder prepared by mixing FNS with 3% Ca(OH)_2_ and 0.03% TEA and grinding it for 81 min also meets the S95 standard for slag powder. The larger dosage of FNS in cement is supported by the observed synergy between TEA and Ca(OH)_2_. This research will provide valuable insights for the expanded application of FNS in construction materials.

## 1. Introduction

Electric furnace ferronickel slag is the solid waste produced during the smelting process of nickel metal or ferronickel alloys in electric furnaces. This slag is generated through water quenching, and its chemical composition is influenced by factors such as raw ore type and smelting processes. The predominant constituents of this slag are MgO, SiO_2_, Al_2_O_3_, and FeO [1,2,3,4,5,6,7]. The slag contains various minerals, such as olivine, tenacious pyroxene, magnesium peridotite, turbidite, and amorphous minerals [8]. Proper treatment of slag is crucial for sustainable ferronickel smelting given the substantial unit slag emission during the process, which amounts to 12–14 tons, and China’s annual production of ferronickel slag exceeding 30 million tons [9,10,11,12,13,14]. The cement industry is currently facing the challenge of reducing energy consumption and greenhouse gas emissions. One potential solution is to partially replace cement raw materials with ferronickel slag, which would not only resolve solid waste disposal issues in metallurgical industries but also reduce overall energy consumption in cement production, thus contributing to carbon emission reduction [15,16,17].

Presently, an increasing number of investigations are devoted to exploring the utilization of ferronickel slag as a supplementary cementitious material. Han et al. [18] successfully used FNS in the production of building materials, and they found that the activity of FNS is relatively low. When the FNS content is less than 10%, FNS can improve the early compressive strength of the material, but when the FNS content exceeds 30%, it is not conducive to the development of strength. Liu et al. [19] stated that ferronickel slag reduces cement system hydration heat and decreases cement paste porosity, resulting in improved paste structure densification. Combining ferronickel slag with mineral powder is more effective in promoting cement strength development and ensuring the stability of the cement system. Su et al. [20] analyzed the leaching dynamics of heavy metals in ferronickel slag from electric furnaces. They confirmed that it is classified as general industrial waste with non-hazardous attributes. The study found that heavy metal Cd is more soluble in water, while Cu and Ni exhibit enhanced leaching in weak acidic conditions. Additionally, Cr, Pb, and Mn have higher leachability in a reducible state, whereas Zn and Cu have elevated leachability in an oxidizable state. Maragkos et al. [21] used XRD and SEM techniques to analyze the microstructures of polymers made from nickel slag as aggregates. They found that under judicious conditions the resulting gelling materials had compact structures, low water absorption, and a compressive strength of up to 118 MPa. Chen et al. [22] successfully prepared impermeable composite materials using ferronickel slag powder. The study showed that ferronickel slag powder can reduce the porosity of composite material, enhance its resistance to erosion, and make the microscopic structure of the composite material more compact, which improved its impermeability. However, due to the limited reactivity of ferronickel slag, it is necessary to limit its dosage to 10% in the aforementioned cement formulations. In summary, while the integration of ferronickel slag as an auxiliary cementitious material exhibits feasibility, it is encumbered by technical challenges associated with large-scale application. Furthermore, comprehensive investigations into leaching toxicity and safety considerations of the cementitious material at elevated dosages remain imperative.

However, challenges remain, particularly regarding its large dosage usage as an auxiliary cementitious material. Further investigation is necessary due to technical impediments and concerns regarding the leaching toxicity and safety of cementitious materials at significant dosages. The issue has been addressed by stimulating the potential activity of ferronickel slag through a combination of mechanical grinding and chemical additives [23,24]. The resulting FNS micropowder meets the quality of S95 slag powder and is suitable for substantial replacement of cement in cementitious material production. This study analyzes the role and synergistic mechanisms of mechanical grinding and different chemical exciters in stimulating ferronickel slag activity using XRD and SEM methods. This provides a foundation for the preparation of high-grade FNS powder and its extensive admixture resource utilization. In practical applications, this study not only provides a reference for the activation of FNS but also provides a basis for the application of large replacements of FNS in cementitious composites.

## 2. Materials and Methods

### 2.1. Materials

#### 2.1.1. Ferronickel Slag

**Figure 1:**
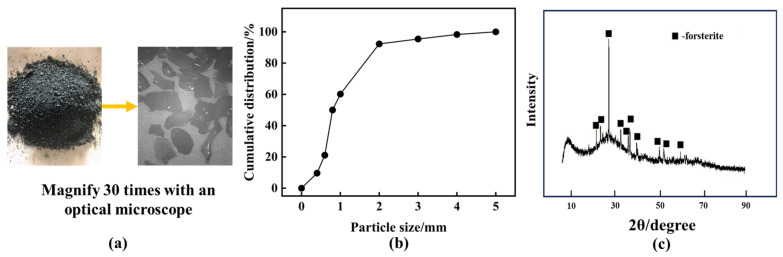
Basic properties of FNS. (**a**) Appearance, (**b**) particle size distribution, (**c**) X-ray diffraction (XRD) pattern.

The ferronickel slag (FNS) utilized in the present study was sourced from Tangshan, Hebei, China, representing a water-quenched and rapidly cooled electric furnace slag. Figure 1 and Table 1 depict the physical morphology, sieved particle size composition, XRD analysis, and XRF analysis results of the FNS. The FNS exhibits a robust texture, uniform particle dimensions, and a dark-green glass-like appearance when exposed to sunlight. Under 30 times magnification with an optical microscope, the surface of the ferronickel slag manifests as smooth, irregularly shaped, and possessing a glass-like luster.

Analysis of particle size composition reveals that 63% of the FNS particles fall within the −1 mm size range, indicative of sufficient water quenching and favoring the original activity of the slag. XRF analysis underscores the prevalence of silicon, magnesium, iron, and aluminum as the most abundant chemical elements in the FNS, categorizing it within the SiO_2_-FeO-MgO ternary slag system. Its primary active component is SiO_2_, with CaO and Al_2_O_3_ content exerting minimal influence on the slag’s activity. Notably, the FNS exhibits low levels of K_2_O and Na_2_O, posing no alkaline-aggregate hazards [25]. However, the presence of Cr and Ni metals is a reminder of the potential leaching toxicity.

The principal crystalline mineral in the FNS is identified as hortonolite, characterized by sharp peaks in the XRD diffraction pattern. Despite the high MgO content (37.43%) in the slag, the chemically synthesized state of Mg in the FNS results in a relatively minor impact on cement stability. The XRD diffractograms reveal bulging peak shapes, indicating a substantial vitreous content in the slag, reaching 90.2% [26]. This high vitreous content suggests significant potential activity for FNS.

#### 2.1.2. Other Raw Materials and Additives

The sand used in the tests of ferronickel slag cement mortar was Chinese ISO standard sand; its particle size range and all indicators comply with GB/T 17671-2021 [27]. This study selected ordinary Portland cement (P.O42.5), whose performance indicators comply with the Chinese national standard “General Portland Cement” (GB-175 [28]). The triethanolamine (TEA), sodium hydroxide (NaOH), calcium sulfate (CaSO_4_), and calcium hydroxide (Ca(OH)_2_) reagents used in this study were from a company in Hebei province, and their purity all met the chemical purity standards.

### 2.2. Exprerimental Methods

#### 2.2.1. Preparation of Materials

The preparation method of FNS powder is to use a conventional ball mill after FNS drying (Φ 500 mm × 500 mm, with a speed of 48 revolutions per minute). The mill was loaded with 100 kg of grinding medium, and each grinding cycle involved 5 kg of FNS. Add different amounts of triethanolamine additives and grind at different times.

Preparation of ferronickel slag cement mortar was according to Chinese National Standard GB/T 17671-2021 [27]. The mass mix ratio for the preparation of ferronickel slag cement mortar test blocks is one part cement, three parts standard sand, and half part water. In total, 450 g ± 2 g of cementitious composites, 1350 g ± 5 g of sand, and 225 mL ± 1 mL of water are required for each preparation of the test block. Make a 40 mm × 40 mm × 160 mm prism in the mold and prepare three specimens at once. Put the formed specimen in the standard curing box for 24 h, and then put it in the temperature (20 ± 1 °C) for curing to the specified age.

The method for making ferronickel slag cement slurry is to use a cement slurry mixer. The mixing pot and blades are first wiped with a damp cloth, and 150 mL of water is added to the pot. Then, 500 g of cementitious composites is added to the water for stirring within 5–10 s, stirring at low speed for 120 s, stopping for 15 s, scraping the surrounding cement into the pot, and then stirring at high speed for 120 s. The machine is stopped, and then the cement slurry is poured out and placed in a curing box for curing, the same as the curing method for cement mortar test blocks.

#### 2.2.2. Property Analysis of Materials

The pozzolanic activity and potential hydraulicity force of the FNS powder were tested according to GB/T12957-2005 [29] and GB/T2847-2022 [30], respectively.

Referring to Chinese National Standard GB/T 1346-2011 [31], the setting time, the water requirement of normal consistency, and the stability of the cementitious materials were measured. The fluidity of the mortar was tested according to Chinese National Standard GB/T 2419-2005 [32]. The Ni and Cr ion concentrations in the sand extract were tested according to GB/T30810-2014 [33]. Compressive strength and flexural strength were tested according to Chinese National Standard GB/T 17671-2021 [27], while the data processing adhered to the international standard ISO 679:2009 [34]. The standard deviation for cement mortar compressive strength and flexural strength testing is 10%, and the results for cement mortar compressive strength and flexural strength are accurate to 0.1 MPa. The activity index Ht of the FNS powder was performed according to GB/T 17671-2021 [27] reference Formula (1):

(1) $$H_{t}=\frac{Q_{t}}{Q_{0}}\times 100\%$$

where *H_t_* is the activity index at age *t*, and retaining one decimal, %; *Q_t_* is the compressive strength of cementitious sand mixed with ferronickel slag micropowder at age *t*, MPa; and *Q*_0_ is the compressive strength of net cementitious sand at age *t*, MPa. 

The effect of different excitation methods on the performance of cementitious materials was compared using the activity excitation coefficient *S_t_*. *S_t_* was calculated as

(2) $$S_{t}=\frac{Q_{ta}-Q_{t}}{Q_{t}}\times 100\%$$

where *S_t_* is the active excitation coefficient of the exciter at age *t*, and accurate to 0.1, %; *Q_ta_* is the compressive strength of the cementitious material specimen added with the exciter at age *t*, MPa; and *Q_t_* is the compressive strength of the cementitious material specimen without the exciter at age *t*, MPa.

Chemically bound water pertains to internal water molecules that establish chemical bonds with the hydrated gel within cementitious materials. The quantification of chemically bound water serves as an indicative measure of the extent of hydration products present in these materials. In the experimental procedure, anhydrous ethanol was employed to arrest the hydration process of specimens derived from cementitious materials at distinct stages of maturation. Subsequently, the specimens underwent desiccation in a drying oven set at 60 °C until a constant weight was attained. Following that, the specimens were weighed, subjected to roasting in a muffle furnace at 900 °C for a duration of 180 min, allowed to cool to room temperature, and then reweighed. The chemically bonded water content, denoted as “*W_nc_*”, for the cementitious composite specimens was computed utilizing the prescribed formula.

(3) $$W_{nc}=\frac{\frac{m_{1}-m_{2}}{m_{1}}-L_{B}}{1-L_{B}}$$

(4) $$L_{B}=(1-\beta )L_{C}+\beta L_{S}$$

where *m*_1_ is the mass of the specimen dried to constant weight at 60 °C; *m*_2_ is the mass of the specimen to constant temperature after burning at 900 °C; *L_C_* and *L_S_* are the loss on ignition of cement and FNS powder, respectively; and *β* is the mass fraction of FNS powder in cementitious composites.

#### 2.2.3. Characteristics Analysis

The surface area of ferronickel slag powder was measured by BET surface area measurement on an ometer (FBT-5, Xinggaoweiye, Tianjin, China), and the particle size distribution analysis was performed by using a laser particle-size analyzer (HL5500-Plus, Haixinrui, Beijing, China). The phase analyses of FNS powder and cementitious composites were determined by an X-ray diffraction spectrometer (XRD, Ultima-IV, Rigaku, Tokyo, Japan), employing continuous scanning within the range of 10° to 90° at a scanning speed of 6°/min. Scanning electron microscopy (SEM, HJ-84, Zeiss, Oberkochen, Germany) was employed to scrutinize the micromorphology of the FNS powder and the slurry morphology of the samples at distinct ages. Furthermore, the chemical elements constituting the hydration product C-S-H gel were analyzed, and the calcium-to-silicon (Ca/Si) ratio was determined utilizing energy-dispersive X-ray spectroscopy energy spectrum scanning (EDS).

## 3. Results and Discussion

### 3.1. Ferronickel Slag Used as Cement Admixture

**Figure 2:**
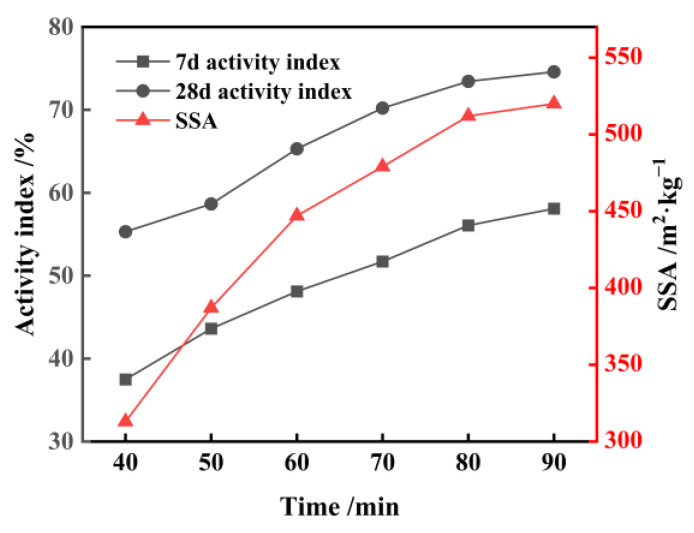
Variation of SSA and activity of FNS with different milling times.

**Figure 3:**
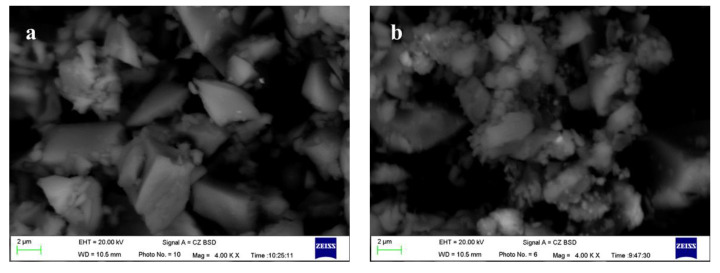
The morphology of FNS powder: (**a**) grinding 60 min, (**b**) grinding 90 min.

Figure 2 shows the changes in specific surface area (SSA), activity index, and micro-morphology of FNS micropowder at different grinding durations. The results indicate a rapid increase in the SSA of the slag powder during the 40–60 min grinding interval, reaching peak grinding efficiency. Subsequently, during the 60–80 min timeframe, the rate of SSA increase decelerates. Beyond 80 min of grinding, a noticeable decline in grinding efficiency is observed, where powder with an SSA exceeding 500 m^2^/kg is obtained. Upon testing, the micropowder with an SSA of 447 m^2^/kg being prepared by grinding for 60 min has a 28 d activity index of 65.3% (≥60%), which meets the requirements of the national standard GB/T2847-2022 [30] for the pozzolanic activity of the cement mixture. The SEM analysis shows that the micropowder particles have a polygonal shape with elongated edges and a smooth surface (refer to Figure 3). When the grinding duration is extended to 90 min, there is a significant increase in the fine particle content within the micropowder, and agglomeration phenomena begin to occur, specifically appearing as small particles clustering spontaneously and adhering to larger particles’ surfaces.

**Figure 4:**
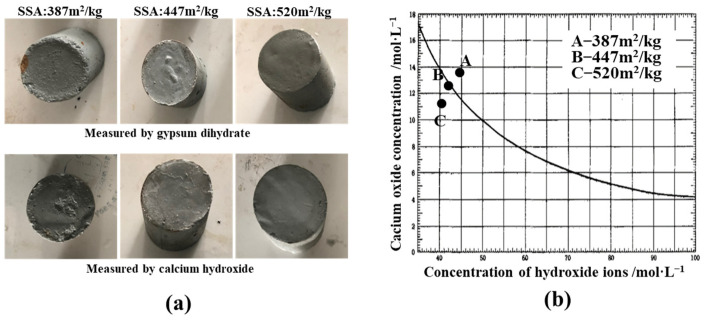
Characterization of FNS. (**a**) Potential hydraulicity, (**b**) pozzolanic activity.

Figure 4 shows the results of the evaluation of the potential hydraulicity and pozzolanic activity of the FNS powder. The results indicate that the powder specimen with an SSA of 387 m^2^/kg deformed and showed evident elevation at the periphery after immersion. In contrast, specimens with an SSA of 447 m^2^/kg and 520 m^2^/kg maintained their intact morphology with clear edges. According to gypsum dihydrate analysis, it is clear that the potential hydraulicity of powder with an SSA of 387 m^2^/kg is not significant, while the micropowders with an SSA of 447 m^2^/kg and 520 m^2^/kg demonstrate favorable potential hydraulicity. Figure 4b illustrates that the Ca(OH)_2_ concentration in the slag with an SSA of 387 m^2^/kg exceeds the calcium hydroxide solubility curve at 40 °C. In contrast, the concentrations of calcium oxide in slags with an SSA of 447 m^2^/kg and 520 m^2^/kg fall below the solubility curve of calcium hydroxide. Therefore, it can be concluded that FNS powder with an SSA of 387 m^2^/kg lacks pozzolanic activity, while the powders with an SSA of 447 m^2^/kg and 520 m^2^/kg meet the criteria for qualified pozzolanic activity.

**Figure 5:**
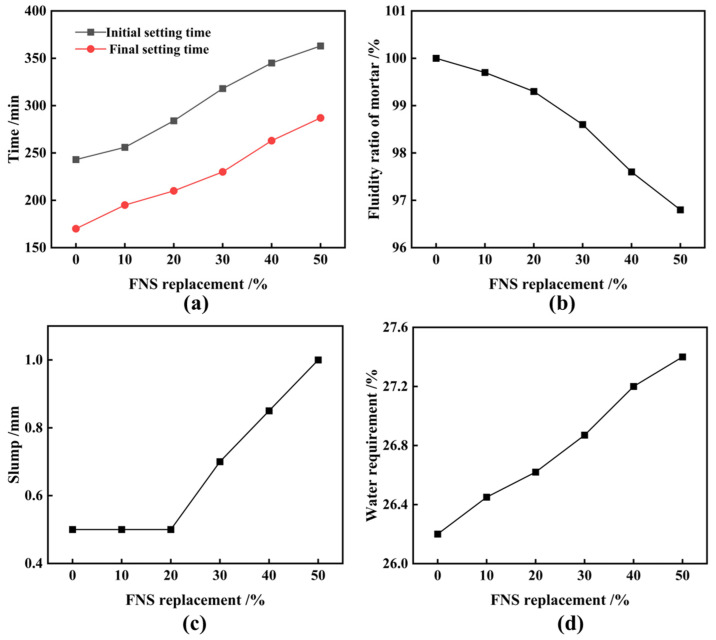
Effect of FNS replacement on cementitious composites’ properties. (**a**) Setting time, (**b**) water requirement for normal consistency, (**c**) stability, (**d**) fluidity ratio.

**Figure 6:**
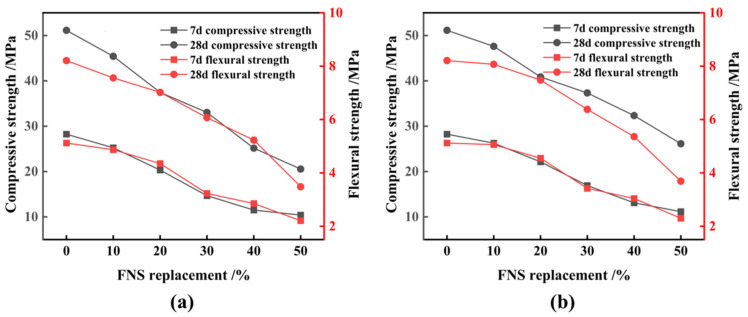
Effect of FNS replacement on strength of ferronickel slag cement mortar. (**a**) 447 kg/m^2^, (**b**) 520 kg/m^2^.

Figure 5 shows the effect of FNS on the cementitious properties of cementitious composites. The water requirement for normal consistency of cementitious composites increases almost linearly with the increasing replacement of FNS, resulting in an increase of approximately 0.2% in water requirement for every 10% increase in FNS replacement. The setting time of cementitious composites also increases proportionally with the replacement of FNS micronized powder. At a 50% replacement, the material’s initial and final setting times measure 287 and 363 min, respectively, meeting the standards outlined in GB/T1346-2011 [31] (initial setting times ≥ 60 min; final setting times ≤ 600 min). Stability tests confirm that all cementitious composite specimens have a swelling distance below 5 mm, indicating a water swelling rate of less than 5% and affirming the qualified stability of cementitious composites. The replacement of FNS in the cementitious material reduces its fluidity, and an excessive amount of FNS further accelerates this reduction in fluidity ratio. This suggests that the fluidity and plasticity of the cement are negatively affected by excessive FNS incorporation (Figure 4). Leaching toxicity tests of ferronickel slag cement mortar showed minimal leaching of Ni and Cr, registering at 0.0635 mg/L and 0.0728 mg/L, respectively, at a 20% replacement, as well as 0.0603 mg/L and 0.1032 mg/L, respectively, at a 50% replacement. These concentrations fall within permissible limits, confirming the absence of leaching toxicity. Figure 6 illustrates the effect of FNS replacement on the strength of ferronickel slag cement mortar. With the increase in FNS replacement, the compressive strength and flexural strength of the cement mortar at different ages are decreased. Generally, FNS replacement lower than 10% does not cause excessive strength loss in the cementitious composites. However, beyond a 20% replacement, the loss of strength accelerates. It is obvious that the effect of FNS powder with an SSA of 520 kg/m^2^ behaved better than the FNS powder with an SSA of 447 kg/m^2^, considering the higher cement mortar strength under the same replacement and the relatively lower decreasing trend in strength with replacement increasing. Consequently, increasing the SSA of FNS is advantageous for maintaining material strength under identical conditions.

### 3.2. Activation of FNS

**Figure 7:**
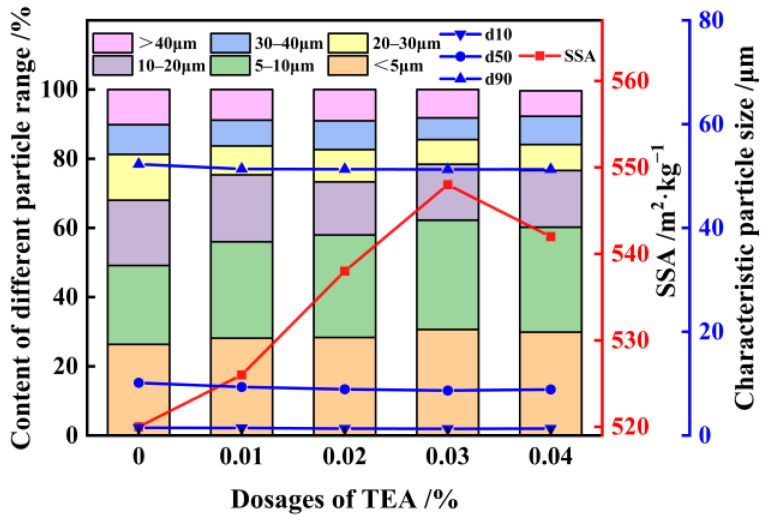
Effect of grinding with TEA additives on the particle-size distribution of FNS powder.

**Figure 8:**
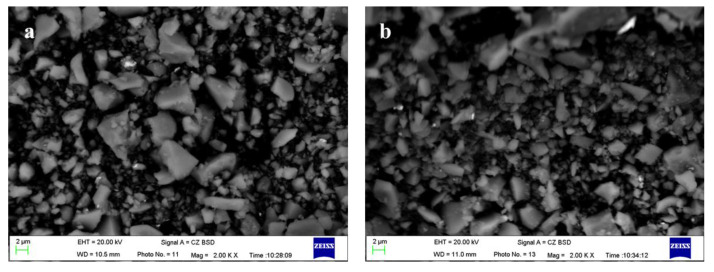
Effect of grinding with TEA additives on the micromorphology of FNS powder. (**a**) 0.03%TEA, (**b**) 0%TEA.

Triethanolamine (TEA) was selected as the grinding aid to conduct tests on FNS [35,36]. The TEA and FNS were thoroughly mixed and milled for 90 min. The results of laser particle-size analysis for FNS micropowder with different TEA dosages are shown in Figure 7. The results show that when TEA is used as a grinding aid, the average particle size of FNS decreases, and there is a significant increase in the percentage of fine-grained particles within −20 μm range. Furthermore, the particle-size distribution of the powder particles follows a more rational pattern. The most significant increase in the SSA of the micropowder occurs at a TEA dosage of 0.03%, resulting in an SSA increase to 548 m^2^/kg. When comparing the scenario with and without TEA-assisted grinding, the content of −5 μm and 5–10 μm particles in the slag powder experiences enhancements of 2.32% and 6.44%, respectively. Figure 8 presents the microscopic morphology of FNS micropowder in the absence of TEA and at the optimal TEA dosage. Notably, the microfine particles in the slag powder with a 0.03% TEA additive exhibit a significant increase, accompanied by a reduction in particle agglomeration. This phenomenon is caused by the interaction of polar groups in the TEA additive with the disrupted ionic bonds on the particle surface. This reduces the particle surface energy and prevents microfine particle agglomeration [37]. Additionally, the TEA adsorption on the particle surface forms a film that acts as a lubricant, allowing for the uniform distribution of FNS particles among the grinding media and consequently improving overall grinding efficacy [38].

**Figure 9:**
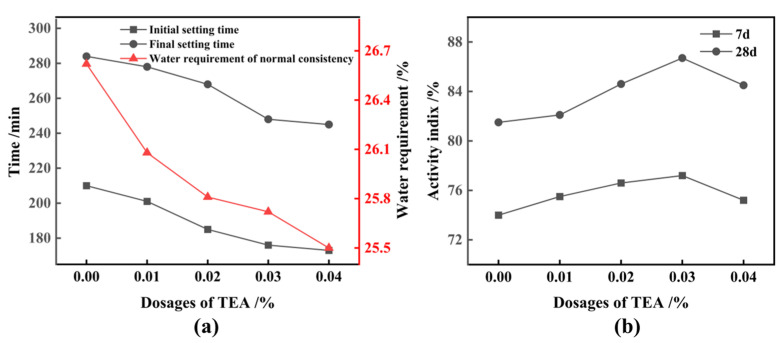
Effect of TEA-aided grinding on the coagulation characteristics and activity index of FNS powder. (**a**) Gel characteristics, (**b**) activity index.

Figure 9 shows the impact of varying dosages of TEA-aided grinding (for 90 min) on the gelling characteristics of cementitious composites and activity index of FNS micropowder, with a fixed FNS replacement of 20%. Evidently, the utilization of TEA additives during grinding markedly reduces the water requirement of normal consistency of cementitious composites. This reduction is attributed to the heightened hydrophilicity of the particle surface induced by TEA additives, leading to a decrease in polymerization forces between particles [39]. The initial and final setting times of the cementitious composites are also diminished, attributable in part to the accelerated cement hydration facilitated by TEA additives and partly to the improved filling effect of microfine slag powder, expediting the overall hydration process [40,41,42]. Furthermore, the 28 d activity index of the slag powder experiences a noteworthy augmentation following TEA-assisted milling. As depicted in Figure 9b, a TEA dosage of 0.03% results in a 6% increase in the 28 d activity index of the FNS micropowder.

**Figure 10:**
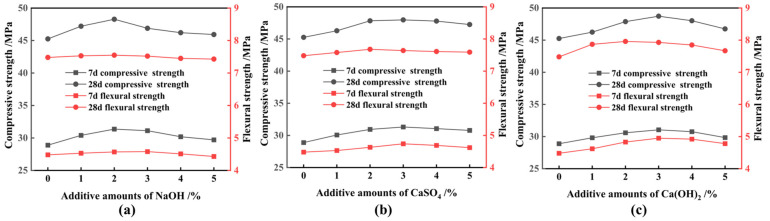
Effect of different chemical elagents on strength of ferronickel slag cement mortar. (**a**) NaOH, (**b**) CaSO_4_, (**c**) Ca(OH)_2_.

**Figure 11:**
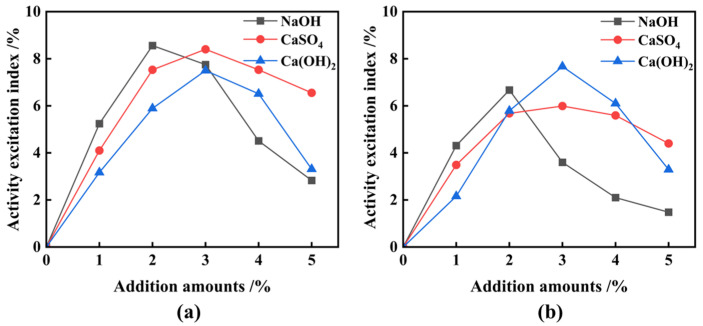
Effect of different chemical elagents on active excitation coefficient. (**a**) 7 d, (**b**) 28 d.

FNS micropowder, ground to an SSA of 548 m^2^/kg through the inclusion of 3% TEA additives, was employed to replace 20% of the cement in equal proportions, followed by chemical excitation; the impact of three chemical excitants on the strength of ferronickel slag cement mortar is illustrated in Figure 10. It is obvious that chemical excitants contribute to enhancing both the compressive and flexural strength of the materials. They have a notable influence on early 7 d strength development, promoting and stimulating it. Their effect on late 28 d strength is even more significant. Figure 11 presents the activity excitation coefficients of the FNS micropowder. Optimal dosages of NaOH, CaSO_4_, and Ca(OH)_2_ were determined to be 2%, 3%, and 3%, respectively. At these optimal dosages, the 7 d activity excitation coefficients for NaOH, CaSO_4_, and Ca(OH)_2_ increased by 9.0%, 8.0%, and 8.0%, respectively, and the 28 d activity excitation coefficients increased by 6.2%, 6.1%, and 7.5%, respectively. A comprehensive evaluation of the effect of exciters on the gelling properties and strength of the composite materials indicated that Ca(OH)_2_ exhibits the most favorable chemical excitation effect. With an additional 3%, the compressive strength of the ferronickel slag cement mortar increased by 2.16 MPa at 7 d and 3.47 MPa at 28 d. Similarly, the flexural strength of the specimen increased by 0.59 MPa at 7 d and 0.55 MPa at 28 d. The 28 d activity index of the FNS micropowder reached 95.8% and met the standard requirements for S95 slag powder. 

**Figure 12:**
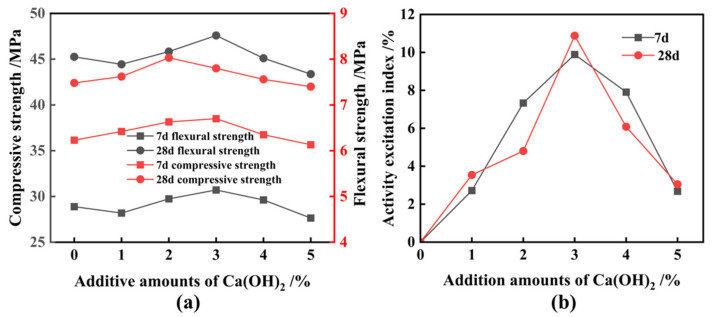
Effect of various Ca(OH)_2_ additions on strength and active excitation coefficient of ferronickel slag cement mortar. (**a**) Strength, (**b**) excitation coefficient.

To expedite the grinding process and reduce energy consumption, a synergistic activation test simultaneously involving TEA and Ca(OH)_2_ was conducted. FNS, 0.03% TEA additives, and 3% Ca(OH)_2_ were combined and ground in the mill for 81 min, resulting in a micropowder with an SSA of 522 m^2^/kg. Following that, test blocks of micropowder were prepared by replacing cement with this micropowder in a 20% equivalent quantity, and the 28 d activity coefficient was measured to be 95.1%, also meeting the standard for S95 slag powder. Initially, 3% of TEA additives and FNS were uniformly mixed and ground for 82 min to obtain a micropowder with an SSA of 524 m^2^/kg. This micropowder was afterwards subjected to chemical excitation using Ca(OH_)2_ to prepare cementitious materials; the relevant performance indicators are presented in Figure 12. The data indicate that the optimal dosage of Ca(OH)_2_ remains at 3%. However, in this case, the 28 d activity excitation coefficient of FNS is only 93.6%, falling short of the standard requirements for S95 slag powder. Hence, the mixing of TEA additives and Ca(OH)_2_ in the grinding stage demonstrates a notable synergistic effect.

### 3.3. Analysis of Synergistic Hydration Mechanism

**Figure 13:**
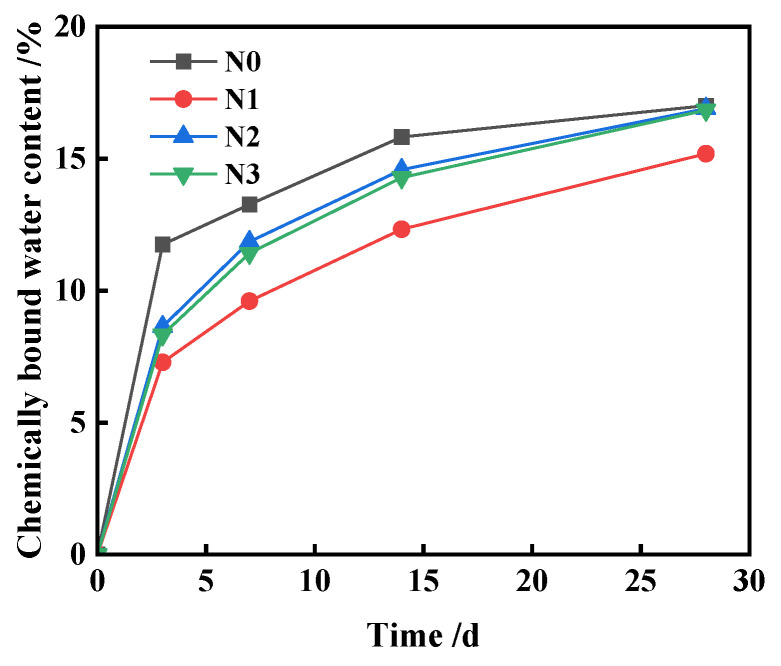
Chemically bound water content of ferronickel slag cement slurry.

Figure 13 presents the chemically bound water content of the ferronickel slag cement slurries, which were prepared with varying additions of TEA additives and Ca(OH)_2_ at their respective optimum dosages. Refer to Table 2 for the specific formula. It is observed that the inclusion of FNS powder reduces the amount of chemically bonded water in the composite cementitious system. Because FNS micropowder is non-cementitious, it struggles to produce enough hydration products in the early stages of hydration. This leads to a notable decrease in the 7 d strength of cementitious composites [1].

**Figure 14:**
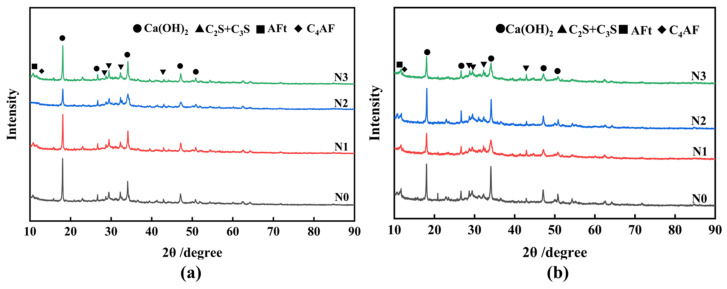
XRD pattern of ferronickel slag cement slurry at different hydration ages. (**a**) 7 d, (**b**) 28 d.

Figure 14 shows the XRD pattern of ferronickel slag cement slurry at different hydration ages. The XRD results show that the main mineral phase compositions in the 7 d and 28 d hydration products of the four groups of blocks are consistent. These compositions primarily consist of cement hydration products, such as calcium hydroxide (CH), calcium aluminate (AFt), calcium ferroaluminate (C_4_AF), and unhydrated minerals C_2_S/C_3_S. However, there is significant variation in the intensity of diffraction peaks for these minerals among specimens. The cement specimens without any additives exhibit the strongest diffraction peaks of NaOH and Ca(OH)_2_ in the 7 d and 28 d hydration products, indicating optimal hydration. However, when 20% unactivated FNS powder is added, the intensity of Ca(OH)_2_ diffraction peaks decreases, while the intensity of C_2_S and C_3_S diffraction peaks slightly increases. This suggests that the presence of FNS reduces the amount of highly hydration-active minerals, thereby slowing down the cement hydration process.

When comparing the XRD spectra of 7 d hydration products from different specimens, it is evident that the negative impact of FNS doping decreases as the SSA of the FNS powder increases. Additionally, TEA additives promote the early hydration of composites, accelerating the hydration reaction rate and increasing the generation of CH. This effect is particularly noticeable for slag powder with a larger SSA. The XRD spectra of the different specimens after 28 d indicate prolonged hydration time, further depolymerization of FNS, gradual secondary pozzolanic reactions, and continuous consumption of CH in the system. As a result, the intensity of CH diffraction peaks in the 28 d specimens is lower. Additionally, an increase in the SSA of the slag powder enhances the secondary pozzolanic reaction of FNS, and TEA additives positively stimulate this secondary pozzolanic activity [43].

**Figure 15:**
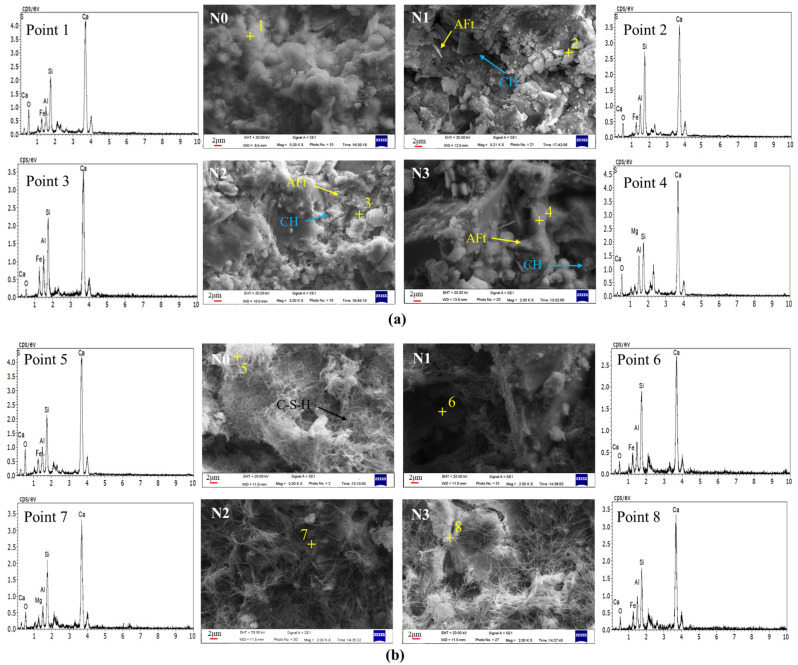
SEM images of ferronickel slag cement slurry at different hydration ages. (**a**) 7 d, (**b**) 28 d.

Figure 15 shows the SEM-EDS image of the ferronickel slag cement slurry, providing insights into the 7 d and 28 d hydration characteristics of different specimens. The 7 d hydration degree of pure cement specimen N0 exhibits superior performance, producing abundant C-S-H gels and layered Ca(OH)_2_ crystals. Additionally, acicular calcium aluminate (C-A-T) gels are observed to grow between voids, tightly surrounding and combining with unhydrated particles. Furthermore, during the 28 d period, the pure cement paste displays a multitude of interlaced hydrated gels with a compact structure.

On the other hand, the 7 d hydrated slurry of N1 exhibits flaky Ca(OH)_2_ crystals and numerous unhydrated particles, resulting in a less dense slurry structure with more pores. Even after 28 d, the hydrated slurry of N1 still contains unhydrated particles, which appear less interconnected with the surroundings, and the reticulated hydration gel remains relatively loose. In the case of the 7 d hydrated slurry of N2, there is a reduction in the number of unhydrated particles and pores, accompanied by an increase in CH content. This results in better-connected hydrated particles. On the 28th day, a hydration gel with a reticulated structure appears, resulting in a denser overall structure with fewer pores that effectively covers the unhydrated particles.

SEM images of hydration products from N0, N1, and N2 show that the increased SSA of FNS micropowder aids in its internal glassy depolymerization. The addition of TEA accelerates the secondary hydration reaction by expediting the alkaline environment around the FNS. The hydrated slurry of test block N2, which was left for 7 d, displays a reticulated and flocculated hydration gel that covers particles and initiates further depolymerization. The slurry densifies after hydration because the finer FNS particles fill fine microvoids. EDS spectroscopy analysis shows a higher calcium content on particle surfaces, which promotes FNS depolymerization and provides sufficient calcium for secondary hydration.

At hydration of 3 d, the slurry of N2 shows clearer reticulation and flocculent hydration gels that tightly connect FNS particles. The intensity of elemental Mg diffraction peaks significantly reduces, emphasizing the prominence of elemental Ca peaks, indicating the coverage of FNS particles by a thick hydration gel. The SEM image of the 7 d hydrated slurry of N3 depicts remaining unhydrated particles with fuzzy edges, suggesting initial depolymerization [23]. After hydration for 3 d, although some particles remain incompletely hydrated, they tightly connect with the slurry surface, demonstrating improved development of hydration products.

Table 3 presents the Ca/Si molar mass ratios of C-S-H gels in the hydration products at various ages of cemented test specimens. The Ca/Si ratio of C-S-H gels in the ferronickel slag cement slurry was observed to be lower than that of pure silicate cement materials at both 7 d and 28 d hydration ages. As the hydration age progressed from 7 d to 28 d, the Ca/Si ratio of C-S-H gel in silicate cement increased, whereas the Ca/Si ratio of ferronickel slag cement slurry decreased. This phenomenon indicates that during the early stages of hydration, the secondary hydration reaction of FNS is relatively weak. The rapid hydration of cement results in the generation of a substantial amount of Ca(OH)_2_. The increased environmental alkalinity subsequently hydrolyzes ferronickel slag, and the depolymerized Si-O and Al-O react with Ca^2+^, continuously consuming Ca^2+^ in the system and reducing the Ca/Si ratio [25,44]. As the cement hydration gradually saturates, the reduced generation of Ca(OH)_2_ in the later stages leads to a decline in the Ca/Si ratio of C-S-H hydrated gel in the ferronickel slag cement slurry. Compared with specimen N1, the Ca/Si of C-S-H gel in the N2 specimen hydrated for 7 d is slightly higher. This is attributed to the initial addition of Ca(OH)_2_, which weakly increases the Ca^2+^ concentration in the system. By the 28 d age, the Ca/Si of C-S-H gel in both N1 and N2 specimens tends to be equal. This observation supports the notion that Ca(OH)_2_ promotes the depolymerization of more FNS particles and facilitates pozzolanic reaction, leading to increased Ca^2+^ participation in the hydration reaction. The judicious reduction of the Ca/Si ratio of C-S-H gel in the ferronickel slag cement slurry contributes to the compaction of the C-S-H gel organization, thereby enhancing the overall strength of the cement.

## 4. Conclusions

This research investigates the impact of grinding time, triethanolamine grinding aid, and chemical excitants on the activation of FNS. The aim is to clarify the mechanism underlying the action of TEA additives and Ca(OH)_2_ in the activation of FNS, as well as their synergistic hydration mechanism. The main conclusions are as follows:

1. Finely ground FNS powder with an SSA of 447 m^2^/kg or more can fulfill the activity requirements of cementitious composites. However, its replacement of cement should be limited to less than 10%. FNS does not compromise stability or exceed leaching toxicity standards. Nevertheless, it does increase the water requirement of normal consistency and the setting time of the cementitious composites while decreasing the material’s fluidity;
2. Ca(OH)_2_ is the best chemical excitation elagent compared to NaOH and CaSO_4_; this is mainly due to it providing a high alkaline environment and amounts of calcium ions during the secondary hydration reaction, and the above-mentioned function is not provided simultaneously by NaOH or CaSO_4_;
3. When FNS is subjected to mixed grinding with 3% Ca(OH)_2_ and 0.03% TEA additives for 81 min, resulting in a micropowder with an SSA of 522 m^2^/kg, which exhibits favorable properties when replacing 20% of the cement. The 7-day and 28-day activities of the FNS micropowder reach 81.0% and 95.1%, respectively, which is comparable to the activity of S95 slag powder. The FNS and 0.03% TEA additives were firstly ground for 82 min to obtain a micropowder with an SSA of 522 m^2^/kg, followed by activating with the addition of 3% Ca(OH)_2;_ this FNS micropowder, however, cannot meet the standard of S95 slag powder. The synergistic activation effect of combining Ca(OH)_2_ and TEA additives during griding is found to be significant;
4. The investigation into the hydration mechanism indicates that the increase in FNS activity is due to the improved alkalinity of the hydration environment caused by Ca(OH)_2_, the positive influence of TEA additives on the particle size and interfacial characteristics of FNS powder, and the associated synergistic effects of these two, which accelerate the depolymerization of the FNS vitreous body, promote the release of more calcium ions for secondary hydration reactions, and lead to the generation of additional hydration products, resulting in a denser slurry structure and higher strength.

This study discusses the optimal replacement and properties of ferronickel slag as cementitious composites and selects the optimal activation conditions. In practical applications, the influence of temperature and humidity on cementitious composites should also be considered. In the future, we will continue to experiment on these aspects to improve the adaptability of the materials.

## Figures and Tables

**Table 1:** XRF analysis results of FNS/wt%.

Oxide	SiO_2_	Al_2_O_3_	Fe_2_O_3_	CaO	MgO	SO_3_
Content	48.17	4.10	6.89	1.10	37.43	0.07
Oxide	K_2_O	TiO_2_	MnO	Cr_2_O_3_	NiO	Na_2_O
Content	0.05	0.05	1.04	1.25	0.03	0.06

**Table 2:** Formulation of cementitious composites.

Sample Number	Cement Content/%	FNS Replacement/%	SSA/m^2^·kg^−1^	Dosages of TEA/%	Additions of Ca(OH)_2_/%	Grinding Time/min	Adding Way of Ca(OH)_2_
N0	100	0	-	-	-	-	-
N1	80	20	520	0	0	90	-
N2	80	20	548	3	3	90	During grinding
N3	80	20	522	3	3	81	After grinding

**Table 3:** Ca/Si molar mass ratio in C-S-H gels of hydration products from different samples.

Hydration Age	7 d				28 d			
Test block	N0	N1	N2	N3	N0	N1	N2	N3
Test result	2.12	1.83	1.85	1.89	2.38	1.63	1.61	1.53

## Data Availability

Data are contained within the article.
